# Enrichment of low-abundance gene mutation by a combined polymerase and ligase chain reaction

**DOI:** 10.1097/MD.0000000000042543

**Published:** 2025-06-27

**Authors:** Zhiqing Huang, Kaisi Li, Yue Zhang, Jiaying Wu, Qiwen Liu, Zixuan Ni, Yan Sun, Beihong Zheng

**Affiliations:** a Center of Reproductive Medicine, Fujian Maternity and Child Health Hospital, Fuzhou, China; b College of Clinical Medicine for Obstetrics & Gynecology and Pediatrics, Fujian Medical University, Fuzhou, China; c School of Basic Medical Sciences, Fujian Medical University, Fuzhou, China; d Fujian Maternal-Fetal Clinical Medicine Research Center, Fuzhou, China

**Keywords:** combined polymerase and ligase chain reaction, gene enrichment, heat-resistant DNA ligase, low-abundance gene mutations, minority alleles

## Abstract

Detection of low-abundance gene mutations or minority alleles in clinical samples is important and challenging in fields of tumor, infectious diseases, noninvasive prenatal diagnosis, and forensic science. The key to solving this problem is the selective enrichment of the low-abundance gene fragments. In this study, a combined polymerase and ligase chain reaction system based on conventional polymerase chain reaction was developed for the first time by introducing a heat-resistant DNA ligase and a pair of ligation primers that target the mutant site. Three *EGFR* gene mutations (L747_S752 del, G719A, and T790M) were used as the target mutations. Both artificial mixed samples containing 1% of 1 of the 3 *EGFR* gene mutations and tumor samples were used to evaluate the feasibility of the proposed new method. Sanger sequencing results showed the coexistence of the wild-type and mutant alleles when the amplification product was obtained by the combined polymerase and ligase chain reaction. The novel method based on the combined polymerase and ligase chain reaction has good performance in the detection of the 2 EGFR mutations, as the commercial kit. The novel method could be used to effectively inhibit the amplification of the wild-type gene fragment in the sample and selectively amplify the low-abundance gene fragment with a mutant site, allowing the mutation to be subsequently detected more effectively, accurately, and reliably. By using this novel method, the mutant gene present at an initial content as low as 1% could be effectively amplified and accurately detected.

## 1. Introduction

With the accumulation of clinical data and the progression of scientific researches, an increasing number of genetic mutants have been identified as important biomarkers for tumor diagnosis, treatment and prognosis.^[1–7]^ Taking epidermal growth factor receptor (*EGFR*) gene as an example, approximately one-third of NSCLCs (non-small-cell lung cancers), harbor an actionable mutation of *EGFR* gene that can be treated with target therapy.^[8–10]^ So actionable mutation and drug resistance-associated mutation of *EGFR* gene (e.g., L747_S752 del, G719A or T790M and so on) must be detected before target therapy with *EGFR*-tyrosine kinase inhibitors (TKIs, such as gefitinib, erlotinib, and afatinib) in patients with non-small cell lung cancer. However, due to the variations in developmental stage, degree of cellular heterogeneity, remote metastasis and tissue type of specimens provided for testing, gene mutations in tumor samples are typically present at a low abundance. For example, cancerous tissue sections and other clinical specimens containing tumor cells shed from the foci (such as plasma, blood, saliva, and stool) are typically a heterogeneous mixture of cancer cells, normal cells and proteins, making it difficult to specifically isolate the cancer cells.^[1,11,12]^ Therefore, it is important and challenging to selectively detect gene mutations in tumor cells that are present at low abundance compared with normal cells. The key to solving this problem is the selective enrichment of these low-abundance gene mutations.

In the present study, a novel method based on a combined polymerase and ligase chain reaction was proposed to enrich low-abundance genes for the first time. This method can be used to inhibit the amplification of the highly abundant DNA fragments, such as a wild-type allele, and selectively amplify the low-abundance DNA fragments, such as specific mutant alleles, thus greatly improving the ability to detect low-abundance gene mutations. In this study, both artificial mixed samples containing 1‰ of 1 of 3 *EGFR* gene mutations (L747_S752 del, G719A, and T790M) and tumor samples were used to evaluate the feasibility of the proposed new method.

## 2. Materials and methods

### 2.1. The principle of the proposed method for enriching low-abundance gene

**Figure 1.**
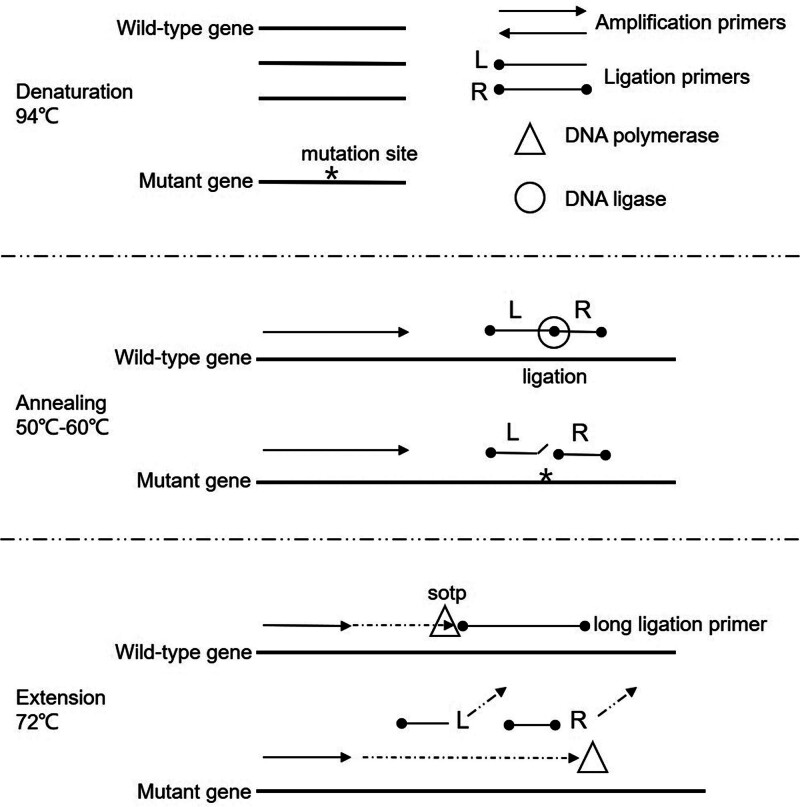
Schematic diagram of the novel method for enriching low-abundance genes. The * on the mutant gene in the diagram represents the mutation site; The left arrows on gene templates represent the amplification primers and their extension direction; L and R represent the ligation primers (the solid dots represent the phosphorylation modification); The triangle represents DNA polymerase, the circle represents DNA ligase; The tilted right end of the L ligation primer on the mutant gene template in the annealing phase indicates that it does not match the mutation site. The dashed arrows extended from the amplification primer in the expansion phase represent the extension of the DNA strand; The inclined dashed arrow in the upper right corner of the ligation primer in the extension phase indicates that the primer is detached from the mutant gene template; The long ligation primer is formed by the ligation primers L and R through DNA ligase.

Figure 1 shows a schematic diagram of the novel method proposed in this study for enriching low-abundance genes. In addition to the use of a heat-resistant DNA polymerase (without 5’-3’ exonuclease activity and strand displacement activity) and an amplification primer pair, which are required for conventional polymerase chain reaction (PCR), the combined polymerase and ligase chain reaction system also contains a heat-resistant DNA ligase and a ligation primer pair. The ligation primer L (the base at the 5’ end of this primer was phosphorylated) and R (the bases at both the 5’ and 3’ ends of this primer were phosphorylated) are completely complementary to the wild-type gene template and are adjacent to each other after binding to the template. The mutation occurs precisely at the junction site. The introduction of a heat-resistant DNA ligase into the amplification system results in the ligation primers L and R being ligated to form a long ligation primer (>40 bp) on the wild-type gene template at the annealing stage (50°C-60°C). However, the ligation primer L is not completely complementary to the mutant gene template at the junction site (i.e., the mutation site), such that the ligation primers L and R are not ligated at the annealing stage for the mutant gene template. When the system enters the next stage (DNA extension at 72°C), because the annealing temperature of the long ligation primer bound to the wild-type gene template is > 72°C, they remain in suit to prevent amplification of the wild-type gene template. In contrast, the annealing temperatures of both short ligation primers L and R bound to the mutant gene are < 60°C; thus, they will both dissociate from the template at 72°C, such that amplification of the mutant gene template is not blocked.

For a tumor sample, if the proportion of wild-type fragment in the target gene is 999/1000, the proportion of the mutant fragment is only 1/1000, and the target gene fragments are exponentially expanded by 2. Assuming that the average inhibition efficiency of the ligation primers for the amplification of the wild-type gene fragment is only 25%, the amplification yield of the wild-type allele is 999 × [2 × (1 − 25%)]^*n*^ (where n is the number of amplification cycles), and the amplification yield of the mutant allele is 1 × 2^*n*^. After 25 cycles, the number of mutant alleles will exceed that of the wild-type alleles. In fact, after optimizing the combined polymerase and ligase chain reaction, the average inhibition efficiency of the ligation primers for the amplification of the wild-type gene template will be over 25%. Thus, the method described in this study will effectively suppress the amplification of the wild-type gene fragment, while the amplification of the mutant gene fragment will be essentially unaffected. Therefore, with an initial mutant gene content in sample as low as 1‰ or even 1‱, this novel method will yield > 50% content of the mutant gene fragment in the final amplification product, allowing subsequent detection of the mutation to be more effective, accurate and reliable.

### 2.2. Construction of low-abundance EGFR mutation system

The G719A (2156 G > C, Cosmic ID: 6239), L747_S752 (2239_2256 del 18, Cosmic ID: 6255), and T790M (2369C > T, Cosmic ID: 6240) mutations on exons 18, exons 19 and 20 of the *EGFR* gene, respectively, were selected to construct *E. coli* DH5α (Takara, Kyoto, Japan) clones containing the mutant gene fragments according to the previously described methods of overlap extension PCR, TA cloning, blue-white screening and Sanger sequencing,^[13,14]^ while simultaneously constructing the *E. coli* DH5α clones containing the corresponding wild-type gene fragments. Subsequently, the positive clones were cultured in liquid LB medium, and the cell density of the clones was determined by a Flow Cytometer. Clones containing the wild-type and mutant gene fragment were mixed at a final ratio of 999:1 to obtain a mixed clones system containing 1‰ of the mutant gene (named mixed sample), with the clone containing only the wild-type gene serving as a control (named sample control). The genomic DNA was extracted from the samples using MiniBEST Bacteria Genomic DNA Extraction Kit (Takara).

### 2.3. Enrichment and detection of the low-abundance EGFR mutation

The combined polymerase and ligase chain reaction system was prepared according to Table 1 and 2, where the DNA template was the genomic DNA from the mixed *E. coli* DH5α clones (mixed sample) or the control clones (sample control, containing only the wild-type gene fragments). Moreover, routine PCR was carried out as the other type of control (Table 1, named reaction control). Healthy human genomic DNA was used as background DNA. The Taq DNA polymerase and heat-resistant Taq DNA ligase were purchased from the company of Takara and NEB (Beverly), respectively. The amplification conditions were as follows: pre-denaturation at 95°C for 5 minutes; 32 cycles of denaturation at 94°C for 20 seconds, annealing at 50°C for 60 seconds, extension at 72°C for 1 minute, and a final extension at 72°C for 10 minutes.

**Table 1:** The combined polymerase and ligase chain reaction system.

Contents	Volume (Test group)	Volume (Reaction control)
10 × PCR buffer	2.5 μL	2.5 μL
dNTP mixture	2 μL	2 μL
Amplification primers (10 μM)	1 μL per primer	1 μL per primer
Ligation primers (20 μM)	0.5 μL per primer	0
10 × ligation buffer	1 μL	0
Taq DNA polymerase	0.125 μL	0.125 μL
Taq DNA ligase	0.2 μL	0
Sample DNA (10 ng/μL)	1 μL	1 μL
Human genomic DNA (10 ng/μL)	1 μL	1 μL
Sterile deionized water	Added to make 25 μL	Added to make 25 μL

PCR = polymerase chain reaction.

**Table 2:** Amplification and ligation primers.

Mutation type	Amplification primers (5’-3’)	Ligation primers (5’-3’)
L747_S752 del	AATATCAGCCTTAGGTGCG	pCGTCGCTATCAAGGAATTAAGA* (L)
	CCAGTAATTGCCTGTTTCC	pGAAGCAACATCTCCGAAAGCCp* (R)
G719A	CAAAAAGATCAAAGTGCTGG	pTTCCAAATGAGCTGGCAAGT* (L)
	GCTCCGGTGCGTTCGGCA	pGTCAATGGCCCCTTTCATAAp* (R)
T790M	ATGCGTCTTCACCTGGAA	pCCACCGTGCAGCTCATCAC* (L)
	GGGACAGGCACTGATTTG	pGCAGCTCATGCCCTTCGGCTp* (R)

* Only the base at the 5’ end of the ligation primer L was phosphorylated, and both the bases at 5’ and 3’ ends of the ligation primer R were phosphorylated. Phosphorylated was marked with the letter p here.

Once low-abundance mutant gene fragments were enriched, some sample molecular biology techniques can be used to detect the gene mutations. The wild-type gene fragment and the mutant fragment could be distinguished according to their features. Sanger sequencing is the golden standard for DNA sequence analysis, so all of the amplification products were submitted to Sangon Biotech Co., Ltd, Shanghai, China) for Sanger sequencing analysis.

### 2.4. Detection of the EGFR gene mutations in tumor tissue and blood

In this study, a total of 37 patients (numbered #1-#37) diagnosed with an advanced stage of NSCLC were enrolled from the affiliated hospitals of Fujian Medical University. According to the *EGFR* gene analysis results of the tumor tissues, all of them were treated with anti-*EGFR* tyrosine kinase inhibitors for the actionable mutations of *EGFR* gene had been detected. Diagnostic biopsy samples (including formalin-fixed and paraffin-embedded [FFPE] samples) before target therapy were collected, and peripheral blood (at least 10 mL) were collected after treatment. Informed consent was obtained from all individual participants included in the study. Ethical approval was obtained from the Scientific and Ethical Committee of Fujian Medical University. Written informed consent was obtained from all patient.

Tumor tissue genomic DNA was extracted from biopsy samples using DNeasy Tissue Kit or from 10 formalin FFPE slides using QIAamp DNA FFPE Tissue Kit (QIAGEN, Dusseldorf, Germany), and cell-free tumor DNA (cftDNA) was extracted from the peripheral blood sample (at least 10 mL) using QIAamp Circulating Nucleic Acid Kit (QIAGEN). After DNA extraction, *EGFR* mutation testing on tissue samples and blood samples of these NSCLC patients were performed using Scorpion-ARMS PCR Kit and Scorpion-ARMS *EGFR* Plasma RGQ PCR Kit (QIAGEN), respectively. All of the relative operation were performed according to each manufacturer protocols. Meanwhile, *EGFR* mutations of all the samples were analyzed by the combined polymerase and ligase chain reaction combined with Sanger sequencing as the method mentioned above.

## 3. Results

### 3.1. Detection result of the artificial mixed samples

**Figure 2.**
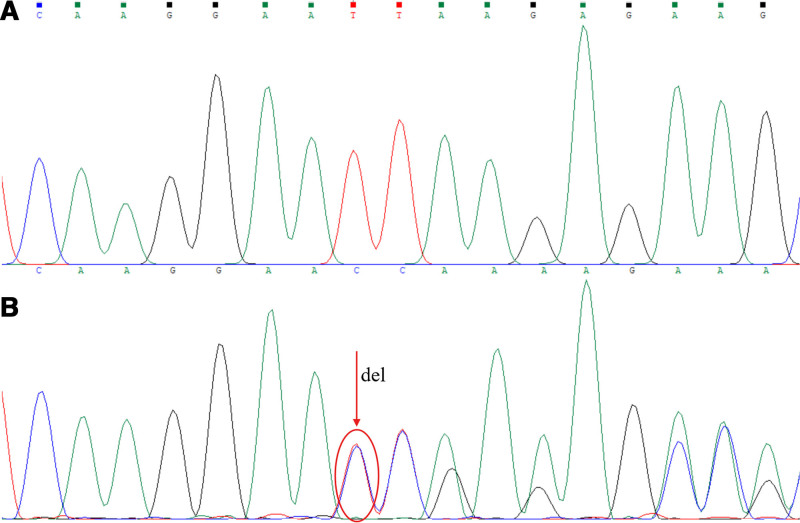
Sanger sequencing profiles for L747_S752 deletion mutation site. (A) corresponding to the amplification fragment of the sample control or the reaction control, and (B) corresponding to the amplification fragment of the mixed clones sample with L747_S752 deletion mutation fragment. The red circle indicates the mutation site, and the red arrow indicates the mutation information.

**Figure 3.**
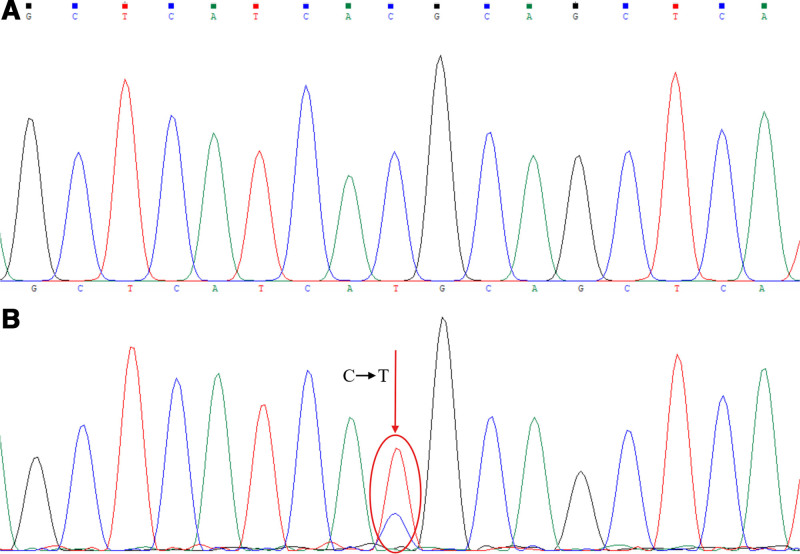
Sanger sequencing profiles for the T790M point mutation site. (A) corresponding to the amplification fragment of the sample control or the reaction control, and (B) corresponding to the amplification fragment of the mixed clones sample with T790M point mutation fragment. The red circle indicates the mutation site, and the red arrow indicates the mutation information.

**Figure 4.**
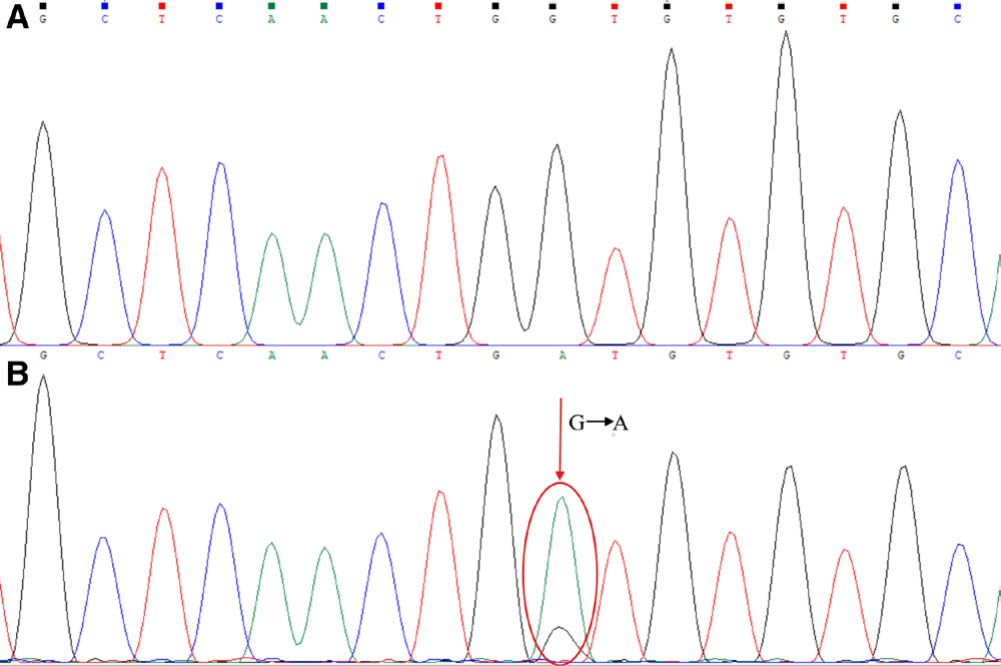
Sanger sequencing profiles for the G719A point mutation site. (A) Corresponding to the amplification fragment of the sample control or the reaction control, and (B) corresponding to the amplification fragment of the mixed clones sample with G719A point mutation fragment. The red circle indicates the mutation site, and the red arrow indicates the mutation information.

The Sanger sequencing results showed only the wild-type gene products were obtained for the sample control and the reaction control (Figs. 2–4). However, for the artificial mixed samples, the Sanger sequencing results showed the coexistence of the wild-type and mutant alleles when amplification product was obtained by the combined polymerase and ligase chain reaction. The L747_S752 del mutation (Fig. 2) exhibited a shifted peak with its beginning at the deletion site, and the T790M and G719A point mutations (Figs. 3 and 4) exhibited double peaks at the mutation sites, all of which are in accordance with the theoretical sequencing profiles of the deletion mutation and the point mutations. In addition, at the mutation sites, the mutation base peaks were high enough to distinguish from the background peaks, even higher than the wild-type base peak, which showed that the amplification products of the mutant gene fragments were more than that of the wild-type gene fragments. Therefore, the Sanger sequencing results confirmed that the combined polymerase and ligase chain reaction could efficiently amplify the low-abundance mutant allele but significantly inhibit the high-abundance wild-type allele, thereby allowing the low-abundance mutant allele (1‰) to be enriched and detected.

### 3.2. Detection result of tumor samples

Then, the novel method was applied to detect the *EGFR* gene mutations in tumor samples (Table 3). L747_S752 was detected in tissue samples of 3 patients (#3, #9, #11) and blood sample of 1 patient (#11) using the Scorpion-ARMS PCR Kit, and the same result was obtained through the novel method proposed in this study, so is the G719A mutation. T790M mutation wasn’t found in the tissue samples no matter what detection method were used. While, T790M mutation was detected in a total of 12 blood samples collected from the NSCLC patients after target therapy (#1, #3, #4, #8, #10, #11-#13, #18, #24-#25, #28) using the Scorpion-ARMS *EGFR* Plasma RGQ PCR Kit or the novel method. So the novel method based on the combined polymerase and ligase chain reaction has the good performance on the detection of the 2 *EGFR* mutations as the commercial kit.

**Table 3:** Result of mutation detection of tumor samples.

Mutation	Scorpion-ARMS PCR Kit	Scorpion-ARMS *EGFR* Plasma RGQ PCR Kit	The novel method proposed in this study	
	Tissue sample	Blood sample	Tissue sample	Blood sample
L747_S752	#3, #9, #11	#11	#3, #9, #11	#11
G719A	#29, #31, #35	#31, #35	#29, #31, #35	#31, #35
T790M	–	#1, #3, #4, #8, #10, #11-#13, #18, #24-#25, #28	–	#1, #3, #4, #8, #10, #11-#13, #18, #24-#25, #28

ARMS = amplification refractory mutation system, EGFR = epidermal growth factor receptor, PCR = polymerase chain reaction.

## 4. Discussion

PCR technology is an important basis for genetic testing. However, conventional PCR technology is not able to selectively amplify the mutant genes in tumor specimens.^[15,16]^ In conventional PCR, the wild-type and mutant alleles are amplified with the same efficiency such that the mutant gene cannot be enriched, making it’s difficult to accurately detect low-abundance gene mutations. Based on the principle of PCR, many new technologies have been recently developed, such as co-amplification at low denaturation temperature-PCR (COLD-PCR), amplification refractory mutation system (ARMS), digital PCR (dPCR) and so on.^[17–22]^ These methods can preferentially enrich low-abundance mutant genes and alleles from a large number of wild-type genes, allowing low-abundance gene mutations and the trace alleles to be accurately detected. These technologies are convenient for sampling, simple to operate, and provide high sensitivity, and thus, are promising for use in a wide range of applications. In addition to tumor-related fields, they can also be applied in noninvasive prenatal diagnosis (such as in the detection of trace fetal alleles in maternal blood),^[23]^ infectious disease diagnosis (such as in the detection of rare HIV drug-resistant gene mutations),^[24,25]^ and forensic science (such as in the detection of specific trace DNA in mixed samples).^[23]^ However, high cost and tedious optimization are usually needed when these method are used.^[13,14]^

In this study, a combined polymerase and ligase chain reaction system based on conventional PCR was developed for the first time by introducing a heat-resistant DNA ligase and a pair of ligation primers that target the mutant site. The method could effectively inhibit the amplification of the wild-type allele in the sample and selectively amplify the low-abundance mutant allele, allowing the mutation to be subsequently detected more effectively, accurately and reliably. In this study, 3 *EGFR* gene mutations (G719A, L747_S752 del and T790M) were used as the target mutations. By using this new method, the mutant gene present at an initial content as low as 1‰ could be effectively amplified and accurately detected. And, this new method can be used to detect the *EGFR* mutation in tumor samples including tissue and blood. In addition, the ligation primers that flank the site of the mutation do not require any other special design and, thus, are very flexible. Once the mutant allele is amplified and the final content exceeds 50%, many available methods can be used for its identification and detection, such as Sanger sequencing, PAGE, endonuclease digestion and so on.^[13,14]^ And a comprehensive system which can detect most of the *EGFR* gene mutations at the same time in 1 tube will be constructed in further study.

In the combined polymerase and ligase chain reaction system, a DNA polymerase without 5’-3’ exonuclease activity and strand displacement activity must be used to allow the long ligation primer to have an inhibitory effect on the amplification of the wild-type allele. A heat-resistant DNA ligase must be used to exert the ligation effect during PCR process. The bases at both the 5’ and 3’ ends of the ligation primer R were phosphorylated so that ligation primer R could be ligated to the ligation primer L and ligation primer R would not be extended by DNA polymerase. For the annealing temperature and primer concentration, both are ligation primer R > ligation primer L > amplification primers, thus ensuring effective ligation and inhibition.

## Author contributions

**Conceptualization:** Zhiqing Huang, Beihong Zheng, Yan Sun.

**Data curation:** Zhiqing Huang.

**Formal analysis:** Jiaying Wu, Qiwen Liu, Zixuan Ni.

**Investigation:** Zhiqing Huang, Kaisi Li, Yue Zhang.

**Methodology:** Zhiqing Huang, Jiaying Wu, Qiwen Liu, Zixuan Ni.

**Supervision:** Kaisi Li, Yue Zhang.

**Writing – original draft:** Kaisi Li, Yue Zhang.

**Writing – review & editing:** Jiaying Wu, Qiwen Liu, Zixuan Ni, Beihong Zheng, Yan Sun.
